# 冷凝集素疾病的诊疗进展

**DOI:** 10.3760/cma.j.issn.0253-2727.2022.06.015

**Published:** 2022-06

**Authors:** 京倩 刘, 凤奎 张

**Affiliations:** 中国医学科学院、北京协和医学院血液病医院（中国医学科学院血液学研究所），实验血液学国家重点实验室，国家血液系统疾病临床医学研究中心，细胞生态海河实验室，天津 300020 State Key Laboratory of Experimental Hematology, National Clinical Research Center for Blood Diseases, Haihe Laboratory of Cell Ecosystem, Institute of Hematology & Blood Diseases Hospital, CAMS & PUMC, Tianjin 300020, China

冷凝集素疾病是冷抗体型自身免疫性溶血性贫血（cAIHA）中的重要类型，临床表现为冷凝集素（CA）介导的溶血性贫血及周围循环症状，包括原发性冷凝集素病（CAD）和继发性冷凝集素综合征（CAS）。随着诊断技术的进步及新药的出现，冷凝集素疾病的诊治有所进展，本文我们就近年来冷凝集素疾病诊治的相关进展综述如下，尤其关注近年来针对B细胞治疗及补体抑制剂治疗相关临床试验。

一、CA特征认识

CA是由B细胞产生的自身抗体，低温时凝集红细胞启动补体介导的溶血[1]。其生理功能始终没有明确，可能为原始脊椎动物免疫系统的残留[2]。

虽然CAD与CAS中都有CA，但两者的CA具有不同特征。首先，产生CA的细胞在CAD和继发于淋巴瘤的CAS中是单克隆的，在继发于感染的CAS中是多克隆的[3]。此外，90％以上CAD患者的CA是对红细胞表面I/i血型抗原特异的单克隆IgMκ，偶有对Pr抗原特异的[3]；而CAS的CA是对I/i特异的IgG或IgM，轻链表型可以是λ或κ[2]。

在CAD中，CA IgM重链85％以上是由14号染色体q臂上的IGHV4-34基因编码，该基因是CA活性的基础[4]。IGHV4-34 FR1对识别I/i抗原至关重要。研究发现，许多患者的IGHV4-34 FR3发生了KLS序列突变，该突变增强了与I抗原结合的能力，而其突变数量与较低的HGB水平相关[4]。除此之外，与I抗原结合的亲和力和特异性还取决于重链互补决定区3（CDR3）和轻链可变区，而轻链多重结合序列的细微差异可能导致热振幅和临床表型的差异[4]。80％ CAD患者的κ轻链是由IGKV3-20或IGKV3-15基因编码，这些基因也和I/i抗原的识别结合有关，且大部分IGKV3-20重排患者表现出高度同源的CDR3区域，这对研发针对CAD的抗免疫球蛋白轻链疫苗提供了基础[4]。

二、冷凝集素疾病特点及分类

既往的许多文献将CAD和CAS混淆使用，而实际上CAD与CAS有所不同。

CAD年发病率约为1/100万，占所有自身免疫性溶血性贫血（AIHA）的13％～15％，是一种具有独特病理和遗传特征的淋巴增殖性疾病（LPD），疾病状态常表现为慢性持续性[1]。1997年Berentsen等[5]在13例CAD患者中发现了克隆性证据，CAD被认为是一种单克隆LPD。随后CAD一度被认为是淋巴浆细胞淋巴瘤（LPL），边缘区淋巴瘤（MZL）等克隆性LPD及华式巨球蛋白血症等单克隆免疫球蛋白疾病。2014年Randen等[6]研究了54例CAD患者的骨髓标本，在这些B细胞中，未发现浆细胞样细胞学特征或浆细胞分化相关转录因子MUM1、XBP1和BLIMP1的表达，少数的成熟单克隆IgM^+^、IgD^−^浆细胞存在于淋巴结节外，并散布在整个骨髓中。未检测到典型的淋巴浆细胞性淋巴瘤MYD88 L265P突变。除1例患者外，未显示BCL6内含子1的突变，这表明淋巴瘤细胞在生发中心尚未成熟。该组病例标本表现出均一的组织学和免疫表型特征，不同于已知的任何一种LPD。2018年Malecka等[7]通过外显子测序等方法检测了16例CAD患者的骨髓样本，检测到淋巴浆细胞淋巴瘤中未显示的KMT2D突变（69％）和CARD11突变（31％），提示KMT2D和CARD11突变可用于CAD的诊断以及与淋巴浆细胞淋巴瘤鉴别，还可作为靶向治疗的基础。这些证据都强烈表明CAD是与LPL、华式巨球蛋白血症等不同的实体。由于CAD是一种惰性疾病，不会发展为全身性淋巴组织增殖性疾病，故将其称为“原发性CA相关性LPD”，与继发于淋巴瘤的CAS相区别[5]。Cesana等[8]建议将少数存在单克隆IgM介导的临床症状但没有骨髓LPD证据的CAD在临床上诊断为IgM相关疾病。在CAD患者中发现最早的细胞遗传学异常是完全或部分3号染色体三体（+3或+3q）。在最近一个包含了12位CAD患者的研究中发现，所有样本都有+3或+3q，大多数患者显示出12号或18号染色体三体，而18号染色体三体的患者治疗反应更差，猜测可能与抗凋亡基因BCL2的上调有关[9]。Berentsen等[10]认为可将BCL2抑制剂venetoclax试用于对治疗没有反应的患者。

CAS较少见，常继发于肺炎支原体、流感病毒感染及恶性肿瘤、实体瘤、自身免疫性疾病等，临床表现取决于原发病，可为一过性或持续性溶血。CAS引起的溶血常在原发病控制后明显改善，这一点明显不同于CAD[2]。随着COVID-19在全球范围内的流行，继发于COVID-19的CAS有多篇报道，猜测可能与COVID-19导致的异常自身免疫和细胞因子风暴有关[11]–[13]，也有接种COVID-19疫苗后CAS加重的报道[14]，具体机制仍不清楚。

温冷抗体混合型AIHA（mAIHA）是AIHA中的一种特殊类型，因为也存在CA介导的溶血，有时可与冷凝集素疾病混淆。mAIHA可原发或继发于其他疾病，患者通常具有高TA（>30 °C）CA，直接抗人球蛋白实验（DAT）对IgG和C3均呈阳性，溶血通常更严重，较少出现冷凝集素疾病常见的微循环症状，初治治疗的mAIHA患者对糖皮质激素反应良好，但极易复发，并常发展为难治性AIHA[15]。冷凝集素疾病患者的CA的TA大多≤30 °C，DAT对C3呈强阳性，通常对IgG呈阴性，也有极少数患者对IgG呈弱阳性，90％以上的患者除溶血外可出现寒冷引起的肢端发绀、网状青斑、雷诺现象等微循环症状，通常对糖皮质激素反应差[16]。

三、治疗

CAD治疗包括非药物治疗和药物治疗，轻症患者通过保暖、输液加温、防治感染等非药物治疗即可改善[10],[17]，非药物治疗效果不佳、症状性贫血、输血依赖或出现严重影响生活的循环系统症状时可选择药物治疗。药物治疗包括一般治疗、针对B细胞的治疗以及补体抑制剂的治疗。一般治疗包括糖皮质激素、细胞毒药物、脾切除术等，疗效较差[10]。针对产生CA的B细胞的治疗为源头治疗，优点是对溶血及微循环症状均有效，缺点是起效较慢，对急性溶血控制不佳。补体抑制剂起效快，CAD及CAS均有效，可用于急性溶血，需长期维持治疗，且无法改善红细胞凝集所致的周围循环症状。

（一）针对B细胞的治疗

利妥昔单抗（RTX）是一种人源化、嵌合的单克隆CD20抗体，为CAD一线治疗方案。在两项前瞻性研究中[18]–[19]，得到的有效（OR）率为45％、54％，完全缓解（CR）率5％、3％，观察到的中位缓解持续时间为6.5个月、11个月。最近的一项包含104例CAD患者的RTX单药治疗的回顾性研究中发现，OR率为59％，39％的患者对重复使用RTX有反应，中位反应持续时间为15个月[20]。RTX单药治疗CAD的CR率虽然低，缓解持续时间短，但安全性良好，无效和复发的患者多次使用仍有效，可基于此开展更有效的方案。另外观察到一些患者治疗后骨髓组织学缓解和临床缓解不一致，确切的机制仍需进一步的研究[19]。

Berentsen等[21]尝试应用RTX-氟达拉滨（Flu）方案治疗CAD，在一项前瞻性多中心试验中纳入了29例CAD患者，OR率为76％，CR率为21％。OR者的中位HGB增加31 g/L，中位缓解持续时间超过66个月，RTX单药无效患者治疗后70％（7/10）发生反应。2020年Berentsen等[20]对29例患者再评估发现，OR率为62％，CR率为38％，中位缓解持续时间77个月，估计5年持续缓解率为71％，较2004年有更好的CR率，且缓解持续时间长，但31％的患者在随访期间出现了恶性肿瘤。RTX-Flu方案较RTX单药治疗的缓解质量和缓解时间更好，对RTX单药无效者也有效，但3～4级（41％）和4级（14％）血液学毒性发生率较高，有继发恶性肿瘤风险，对于高龄、合并症较多的患者仍需权衡风险利弊。该试验显示，坚持Flu的预定剂量与应答水平没有关联，一些研究发现减低RTX剂量后疗效没有明显下降[22]，这些都使得进一步优化RTX+Flu组合的剂量成为可能。

苯达莫司汀常用于B细胞淋巴瘤的治疗。2017年，在Berentsen等[23]一项前瞻性非随机多中心试验中，45例CAD患者接受了苯达莫司汀和RTX联合治疗后，OR率71％，CR率40％，OR者中位HGB增加了40 g/L，91％的患者至随访32个月时仍处于持续缓解状态。其中，33％的患者出现3～4级中性粒细胞减少症，但只有11％出现感染。2020年Berentsen等[20]对45例患者的再评估发现，OR率78％，CR率53％，随访至88个月仍未达到中位估计缓解持续时间，估计5年持续缓解率为77％，较2017年的OR率和中位反应持续时间更佳，有9％的患者在随访期间发生了恶性肿瘤。该方案与RTX-Flu组相比，OR率相似，但CR率更高，反应持续时间更长，有更好的安全性，对RTX单药或RTX-Flu无效的患者也有效，可作为CAD患者的治疗选择之一。

硼替佐米是26S蛋白酶体抑制剂，通过阻断细胞内多种调控细胞凋亡及信号传导的蛋白质的降解起作用。在Rossi等[24]的Ⅱ期GIMEMA研究中，短暂的硼替佐米疗程OR率和CR率分别为32％和16％，三分之二的患者在中位随访16个月后仍保持反应。该疗法对RTX单药和细胞抑制剂等治疗失败的患者有效，且缓解持续时间长，治疗耐受性好，毒性有限，这些数据为进一步研究更好的治疗方案或与其他药物的组合使用成为可能。

BTK抑制剂对继发于CLL和套细胞淋巴瘤的AIHA疗效较好（主要为温抗体型）。2020年，方力维等[25]将伊布替尼用于2例复发难治性mAIHA患者，420 mg/d伊布替尼2周后HGB增长均>20 g/L，分别在使用6周和10周后HGB>110 g/L，且维持疗效10周以上，疗效显著。2022年，一项包含4例CAD和9例CAS的伊布替尼的临床研究发现，420 mg/d伊布替尼治疗3月得到的CR率77％，治疗后12个月CR率92％，反应最佳者HGB中位增长56 g/L，11例输血依赖患者均脱离输血，9例有周围循环症状患者均有改善，1例患者发生3级中性粒细胞减少（7.7％）[26]。伊布替尼不仅可以快速改善溶血性贫血，还对周围循环症状有效，对复发难治的CAD患者不失为一种选择。

达雷妥尤单抗是针对浆细胞和淋巴浆细胞表面跨膜糖蛋白CD38的单克隆抗体，已证明对MM有效。2020年[27]和2021年[28]各报道了一例达雷妥尤单抗在复发难治的CAD患者中取得良好疗效的病例，在经过8个周期和19个周期达雷妥尤单抗后患者HGB均增长>30 g/L，溶血及循环症状消失，脱离输血，其中一例患者观察到CA滴度的下降和骨髓改善，这为达雷妥尤单抗用于CAD患者提供了依据。

（二）补体抑制剂

经典补体途径介导的血管外溶血被认为是CAD稳定期主要溶血机制。寒冷时CAD患者的CA结合RBC形成CA-RBC复合物，与C1q结合启动补体经典途径，顺序活化C1r、C1s、C4、C2生成C4b2a（C3转化酶），裂解C3为C3a和C3b。补体C3b致敏的RBC通过肝网状内皮系统时被巨噬细胞吞噬发生血管外溶血，大量补体消耗限制了溶血速率。而C3b结合C4b2a形成C4b2a3b（C5转化酶）裂解C5，导致膜攻击复合物（MAC）的形成，故15％的患者也可观察到血管内溶血。在高热、感染或重大手术等急性期，大量补体蛋白产生和末端途径激活，主要表现为血管内溶血[29]。

鼠源性单克隆抗体TNT003选择性靶向作用于C1s，仅抑制补体经典途径，保留了正常的免疫监视及C1q的调理功能（即凋亡细胞碎片的吞噬清除），TNT003在体外实验中以浓度依赖的方式抑制溶血[29]。Sutimlimab（BIVV009/TNT009）是以TNT003为基础出现的靶向C1s的人源化单克隆抗体，在其Ⅰb期临床实验中，10例CAD患者接受Sutimlimab治疗后第1周HGB中位增长16 g/L，6周后70％患者HGB增长>20 g/L，50％患者HGB增长>40 g/L，未观察到药物相关不良反应[30]。在其为期26周的多中心、开放、单臂Ⅲ期临床试验中，用药第1周就观察到溶血的控制、疲劳减轻及生活质量改善，在第23、25和26周治疗评估时，患者HGB平均值（最小二乘法）增加26 g/L，患者第3周起HGB平均保持在110 g/L以上，71％的CAD患者未接受输血（5～26周），未见到药物相关的严重不良事件[29]。Sutimlimab因治疗冷凝集素疾病中良好的数据被FDA授予突破性药物资格（BTD）和孤儿药资格（ODD），如果获批上市，Sutimlimab将成为第一个治疗CAD溶血的药物。

ANX005是一种抑制C1q的人源化单克隆抗体，在体外实验中可防止C4和C3沉积于CAD血清中的人红细胞上，呈剂量依赖性。在绵羊红细胞实验中，ANX005可以阻止经典补体激活以及溶血[31]，这为其在CAD中的临床应用提供了依据，ANX005在健康志愿者进行的安全性实验数据仍未公布。

C1-INH是一种补体调节蛋白，可以通过抑制C1r、C1s等的活性抑制补体经典途径，控制溶血。Wouters等[32]应用C1-INH治疗AIHA，成功预防输血时溶血的加重，然而CAD患者可持续正常产生C1-INH，控制溶血需要持续较大剂量的C1-INH，且停药即可复发，这些都限制了该药物在CAD患者的临床应用。

APL-2是一种坎普他汀类似物，一种聚乙二醇化的环肽，可通过阻止C3b产生预防血管内和血管外溶血的作用，临床前的研究发现，其皮下给药的途径具有良好的药代动力学[33]。两项双盲、安慰剂对照的单中心研究发现，最早在给药开始后八天就显著降低了溶血活性，并且在给药期间一直保持这种抑制作用，且健康志愿者的安全性研究尚未显示出感染风险，但经验有限，仍需更多数据[34]。在其与Eculizumab头对头的Ⅲ期对照研究中，APL-2在改善PNH患者血红蛋白水平，临床和血液学结果方面优于Eculizumab，且消除了Eculizumab导致的C3在红细胞上的沉积所致的溶血[35]。作为一种有希望的新型补体靶向治疗，APL-2有望应用于冷凝集素疾病患者。

Eculizumab是补体C5的长效人源化单克隆抗体。它通过与C5结合防止过敏毒素C5a和MAC的产生，可用于CAD的急性加重。该药物已在美国和欧盟申请批准用于PNH。一项包括12例慢性CAD患者和1例急性CAS患者的前瞻性临床试验显示，9例输血依赖患者经Eculizumab治疗后8例脱离输血，1例输血需求减少，但寒冷引起的循环症状以及生活质量没有改善，且TA较宽的CA引起溶血的患者需要更高的剂量[36]。在稳定的冷凝集素疾病患者中，C5介导的血管内溶血可能并不明显，但在患者高热、感染、创伤或大手术期等的急性溶血发作中发挥重要作用。急性CAS患者在输注红细胞后出现严重血管内溶血，在给予Eculizumab后24 h内，血红蛋白尿停止，患者康复[36]。急性溶血的阵发性冷性血红蛋白尿（PCH）患者在给予单剂Eculizumab后溶血快速得到控制，在随访的4周内HGB升至113 g/L，未出现药物不良反应[37]。Eculizumab控制血管内溶血非常有效，Notaro等[38]研究发现Eculizumab在消除PNH患者血管外溶血时可由于C3d在红细胞的沉积，使得PNH患者经历轻至中度血管外溶血，而APL-2可消除这种影响[35]。类似情况还未见于CAD，仍需更多研究证实，而老年患者应用时受到药物毒性的限制，使用时需权衡利弊。

四、小结

随着研究的深入，对于冷凝集素疾病的认识越来越清晰。以RTX为基础的联合治疗、蛋白酶体抑制剂、BTK抑制剂、补体抑制剂等都取得较好的进展，但疾病缓解时间短、需长期用药及复发率高等仍是今后临床要长期面对的问题，而CAD组织学缓解和临床缓解不同步的原因以及CAS的治疗仍需要更多的研究。
